# Direct comparison of coronary microvascular obstruction evaluation using CMR feature tracking and layer-specific speckle tracking echocardiography in STEMI patients

**DOI:** 10.1007/s10554-023-02998-5

**Published:** 2023-11-13

**Authors:** Chaofan Wang, Lili Wang, Jie Yin, Haochen Xuan, Junhong Chen, Dongye Li, Xiancun Hou, Tongda Xu

**Affiliations:** 1 Department of Cardiology, The Affiliated Hospital of Xuzhou Medical University, Xuzhou, Jiangsu People’s Republic of China; 2 Department of Cardiology, The Second Affiliated Hospital of Xuzhou Medical University, Xuzhou, People’s Republic of China

**Keywords:** Coronary microvascular obstruction, Magnetic resonance feature tracking, Speckle tracking echocardiography, ST-segment elevation Myocardial Infarction

## Abstract

**Purpose:**

Layer-specific speckle tissue echocardiography (LS-STE) is a unique technique used to assess coronary microvascular obstruction (CMVO) that may offer more information on the myocardial anatomy of patients with ST-elevation myocardial infarction (STEMI). Cardiovascular magnetic resonance feature tracking (CMR-FT) has also been gaining popularity as a way to evaluate CMVO. The aim of the present study was to directly compare CMVO assessment in STEMI patients using CMR-FT and LS-STE.

**Patients and methods:**

A total of 105 STEMI patients with LS-STE, CMR-FT, and primary percutaneous coronary intervention (PPCI) were included in the study. Longitudinal peak systolic strain (LS), circumferential peak systolic strain (CS), and radial peak systolic strain (RS) were each used to evaluate CMVO using CMR-FT and LS-STE.

**Results:**

Correlation coefficients were 0.56, 0.53, and 0.55 for CMR-FT CS vs. endocardial CS, midcardial CS, and epicardial CS comparisons, respectively, and 0.87, 0.51, and 0.32 for CMR-FT LS vs. endocardial LS, midcardial LS, and epicardial LS comparisons, respectively. Bland-Altman analysis revealed strong inter-modality agreement and little bias in endocardial LS, while the absolute of limited of agreement (LOA) value was 2.28 ± 4.48. The absolutes LOA values were 1.26 ± 11.16, -0.02 ± 12.21, and − 1.3 ± 10.27 for endocardial, midcardial, and epicardial respectively. Intraclass correlation coefficient value of 0.87 showed good reliability in endocardial LS, and moderate reliability with values of 0.71, 0.70, and 0.64 in endocardial, midcardial, and epicardial CS, respectively (all *p* < 0.001).

**Conclusions:**

CMR-FT is a viable technique for CMVO evaluation in STEMI patients. Endocardial LS showed good reliability for CMR-FT. STEMI patients can undergo LS-STE to assess the CMVO before PPCI.

**Supplementary Information:**

The online version contains supplementary material available at 10.1007/s10554-023-02998-5.

## Introduction

The goal of increasing early survival following ST-elevation myocardial infarction (STEMI) has been accomplished in the last few decades [1, 2]. Following the risk stratification procedures is a prerequisite for treatment strategy guidance for diseases like diabetes mellitus and hypertension [3]. Coronary microvascular obstruction (CMVO), intra-myocardial hemorrhage, and intra-myocardial edema also affect the prognosis of the patients with STEMI [4]. The importance of the coronary microvascular system for STEMI prognosis has long been underemphasized. It is one of the major components of the coronary circulation. The efficiency of myocardial revascularization may be hampered by the presence of CMVO with STEMI. In about 30% of patients undergoing primary percutaneous coronary intervention (PPCI) with STEMI, CMVO is to blame for the persistence or recurrence of angina and even heart failure [5]. There are still conflicting results that complete revascularization is better than intensive medical therapy alone in some patients.

It has been shown that CMVO can forecast clinical outcome and post-infarction left ventricular remodeling [6]. Therefore, how to evaluate CMVO has become the problem that needs to be solved immediately. Patients’ clinical presentation and the existence of comorbidities influence the drug treatment methods. An invasive coronary flow reserve (CFR) test is a gold standard used to evaluate functional disorders of coronary microcirculation [7]. It is not widely performed in clinical work due to its complexity, especially in STEMI patients. Coronary microcirculation cannot yet be directly morphologically observed using any imaging modality. However, there are many noteworthy non-invasive tests such as transthoracic Doppler echocardiography, positron emission tomography and cardiovascular magnetic resonance (CMR) [8].

CMR is a non-invasive clinical tool for the assessment of the STEMI patients [9]. Myocardial perfusion can also be evaluated using late gadolinium enhancement (LGE). Its excellent resolution makes it possible to see transmural myocardial flow and is regarded as the gold standard for non-invasively measuring CMVO [10]. Recent research has demonstrated that cardiovascular magnetic resonance feature tracking (CMR-FT) may accurately measure myocardial strain without the requirement for customized pulse sequences or additional scanning time [11].

Speckle tissue echocardiography (STE) is widely used in clinical practice. Myocardial strain, which includes longitudinal peak systolic strain (LS), circumferential peak systolic strain (CS), and radial peak systolic strain (RS), is a quantitative measure of myocardial deformation [12]. Layer-specific STE (LS-STE) analysis has advanced thanks to novel developments in strain imaging techniques. It is utilized for the study of cardiac distortion in clinical practice. LS-STE is a simple, affordable, and painless technique [13]. It may open the door to earlier pathology detection and disease localization in STEMI patients.

The two techniques both have their advantages in detecting the CMVO. Tamarappoo et al. had reported that CMR-FT can predict CMVO as an early sign of LV mechanical dysfunction in STEMI [14]. Garg et al. had demonstrated that STE had the strongest association with CMVO [15]. Strain can also have an impact on how patients with STEMI are categorized in terms of risk. More research is necessary in this area before applying this knowledge in the clinic. CMR-FT is an established technique for predicting CMVO [15]. LS-STE is another method for predicting CMVO due to its versatility, availability, and low cost. In the left ventricle of healthy Chinese participants, Liu et al. discovered that multilayer strain and CMR-FT had a satisfactory connection in circumferential and longitudinal strain in the LV of healthy Chinese participants [16]. Previous studies have reported the prognostic value of CMVO. However, the inter-modality agreement for LS-STE and CMR-FT has not been evaluated, and direct comparison between the novel LS-STE and CMR-FT for CMVO prediction has not been conducted. The present study compares CMR-FT and LS-STE for the assessment of CMVO in STEMI patients.

## Materials and methods

### Study population and design

This present investigation was conducted retrospectively at a single center. A total of 105 patients who underwent PPCI with STEMI, echocardiography, and CMR procedures between 2020 and 2022 were enrolled in the study. All of the patients received PPCI within 12 h of chest discomfort onset and met the diagnostic requirements for STEMI [17]. The worldwide guidelines suggestions were followed for PPCI. A preliminary TIMI of 0 or 1 anterograde flow in the culprit artery and a subsequent TIMI III flow were shown to each of them. All patients ultimately experienced full revascularization. Patients underwent CMR and echocardiography examinations within 5–7 days following the myocardial infarction.

**Fig. 1:**
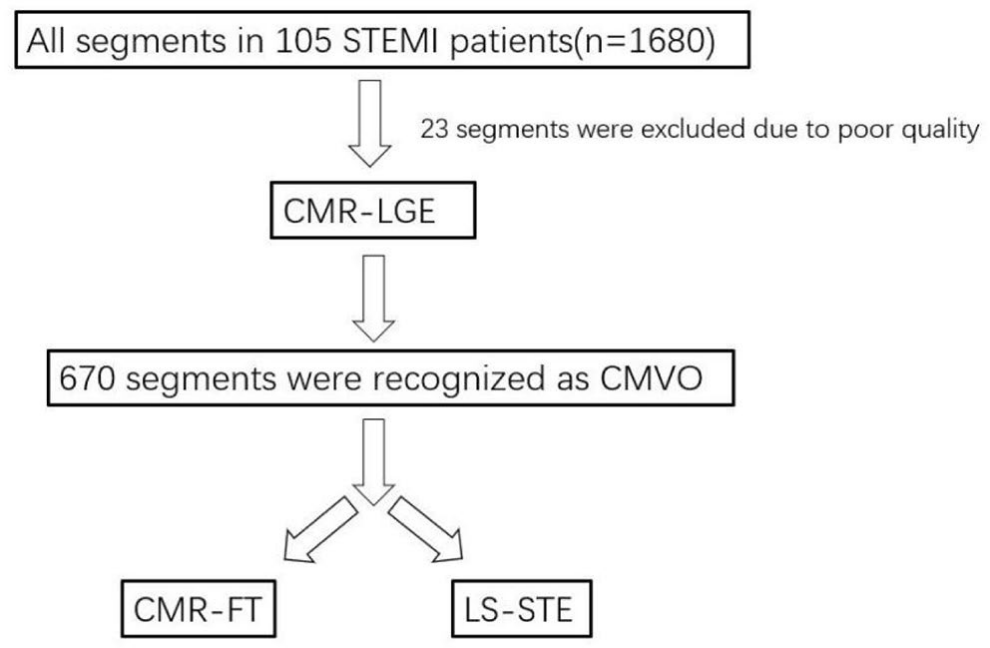
The flow chart of the study. **Abbreviations** STEMI, ST-elevation myocardial infarction

Patients with a history of myocardial infarction, unsatisfactory segmental imaging, and a lack of echocardiography and CMR examinations were excluded from the study (Fig. 1).

### CMR acquisition

All CMR exams were conducted on a 3.0 T magnetic resonance scanner system (Philips Healthcare, Best, The Netherlands) within 5–7 days of successful reperfusion. Cine-CMR tests were carried out while holding breath and were electrocardiogram-triggered. The images were taken in order to evaluate the segmental myocardial function. Then, 8-mm short-axis slices were collected using a prospective electrocardiographically gated gradient-echo sequence with an inversion prepulse after 15 min of intravenous administration of 0.2 mmol/kg gadolinium diethlenetriamine pentacetic acid (Magnevist, Schering, Berlin, Germany).

### CMR-FT analysis

**Fig. 2:**
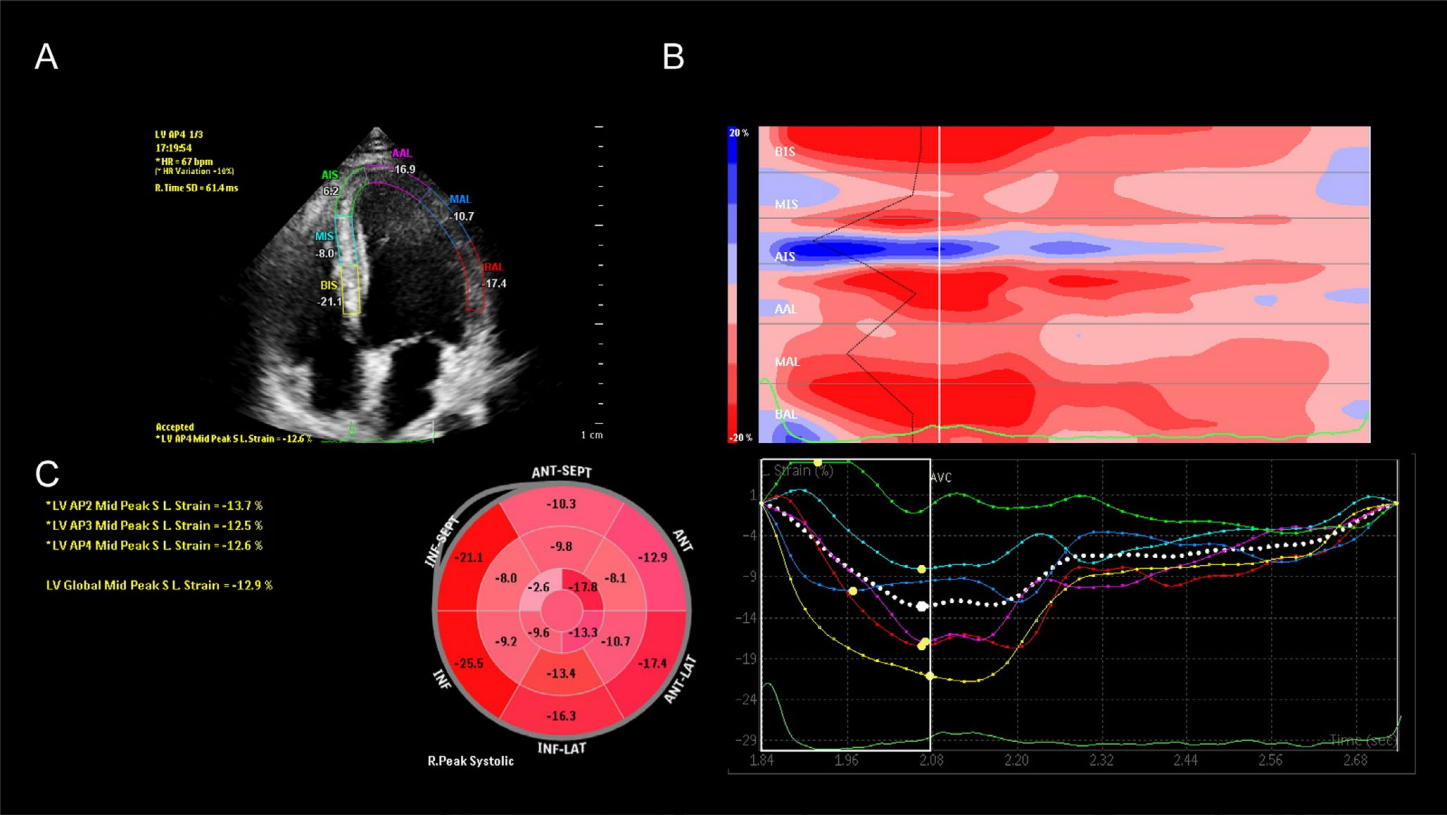
LS-STE and CMR-FT analysis. **Notes**: **A:** Example of a patient the apical four chamber view, measurement of LS-STE. **B-C**: Example of a patient the long axis section, measurement of CMR-FT

Commercially available Cvi42 software system Segment version 2.0 was used to perform a quantitative evaluation of the CMR-FT images (Calgary, Canada). The endocardium and epicardium of the short-axis section of the layer were manually sketched after determining the locations of the left and right ventricles’ insertion points. The endocardium and epicardium of the remaining short- and long-axis sections were then automatically generated using the AI smart key. The endocardium contours of the portions with plainly mismatched contours were manually sketched after the software automatically recognized the endocardium and epicardium contours of the remaining short-axis segment and long-axis section. Finally, strain analysis parameters were obtained. The values of each strain measure, including the LS, CS, and RS, represent the peak strain values in systole (Fig. 2 and Supplementary Fig. 2).

### CMR-LGE acquisition

The collected magnetic resonance delayed enhancement sequence was imported into the cvi42 software. The center of the infarcted myocardium’s low-signal area was classified as a CMVO. The myocardium was divided using a 16-segment model, which was then used as the benchmark to define the myocardial segments for CMVO.

### Echocardiography acquisition and LS-STE analysis

Patients were routinely examined utilizing an S5-1 transducer-equipped commercially available system (Philips IE 33, Philips Medical Systems, Cleveland, OH, USA) while lying in the left lateral decubitus posture. Next, 12-lead electrocardiography and blood pressure readings were taken throughout the evaluation. At the basal, midventricular, and apical levels, two-dimensional grayscale images of the apical four-chamber, apical two-chamber, apical long-axis, and parasternal LV short-axis views were recorded. In order to obtain the images, harmonic (1/3 MHz) B-mode imaging was used at a frame rate of 60–70 frames/s. Three consecutive cardiac cycles were recorded for each view while the patients were holding his or her breath.

The Q-Lab workstation was used to imported the photos (software 8.1 S Germany). The proper myocardial thickness, the mitral annulus, the LV apex, the LV endocardium, and the epicardium were measured simultaneously. The section boundaries were manually modified if they were unsatisfactory. The software also provided speckle tracking results and automatically separated the heart muscle into the endocardium, mid-myocardium, and epicardium. LS, CS, and RS were calculated for three layers in 16 segments during the three cardiac cycles (Fig. 2 and supplementary Fig. 1).

All of the parameters were included in the RS, endocardial CS (EndoCS), midcardial CS (MidCS), epicardial CS (EpiCS), endocardial LS (EndoLS), midcardial LS (MidLS), and epicardial LS (EpiLS).

### Repeatability

Two independent observers used the same echocardiographic and CMR images to determine all of the parameters (inter-observer variability). Similarly, the same observer analyzed all of the images more than three months apart (intra-observer variability).

### Statistical analysis

R Studio software version 4.1.3 was used for all statistical analysis, and a *p-*value of 0.05 was regarded as statistically significant. The mean and standard deviation were used to express continuous data. Bland-Altman plots were used to gauge agreement. Pearson’s correlation coefficient and the intraclass correlation coefficient (ICC) were used to compare the agreements. The mean difference between the CMR-FT and STE datasets was evaluated using the paired sample t-test.

## Results

All patients underwent PCI and their coronary angiography results revealed signs of STEMI. The study cohort is represented in Table 1. There were 1680 LV segments in total, however 23 segments were excluded due to low image quality. CMVO was present in 670 infarcted segments in the 105 patients. CMR and echocardiography results were obtained for all patients. There were 670 segments in total (Fig. 1).

**Table 1:** All patients showed evidence of STEMI and underwent PCI

Clinical data	STEMI patients (n = 105)	%
Age (years)	55.4 ± 12.3	
Male	68	64.76
Hypertension (> 140/90mmHg)	43	40.95
Diabetes mellitus	64	60.95
Total cholesterol (> 5mmol/L)	70	66.67
Smoking	72	70.59
LVEF (%)	49.6 ± 5.3	
Heart rate (bpm)	78.8 ± 11.0	
Time of onset (h)	6.2 ± 2.3	
D to W (min)	74.1 ± 7.1	
Related arteries		
LAD	39	37.1
LCX	23	21.9
RCA	25	23.8
LAD + LCX	10	9.5
LAD + RCA	8	7.6

**Abbreviations**: STEMI, ST-elevation myocardial infarction; PCI, percutaneous coronary intervention; LVEF, Left ventricular ejection fraction; D to W, Door to wire; LAD, Left anterior descending; LCX, Left circumflex; RCA, Right coronary artery

### Direct comparison of CMR-FT and LS-STE to evaluate CMVO

**Fig. 3:**
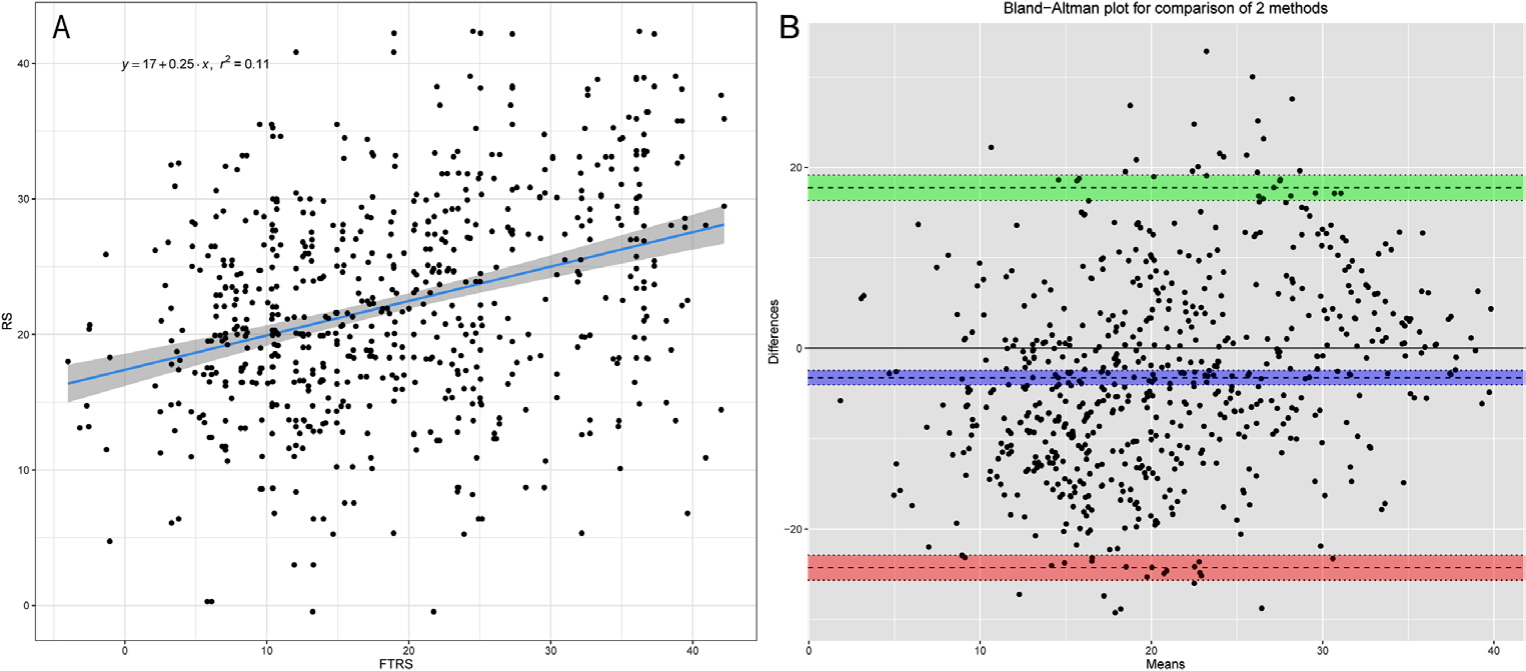
Inter-modality agreement and correlation between CMR-FT and LS-STE derived strain parameters for RS. **A:** Correlation FT-RS vs. RS; **B:** Bland Altman: FT-RS vs. RS

**Fig. 4:**
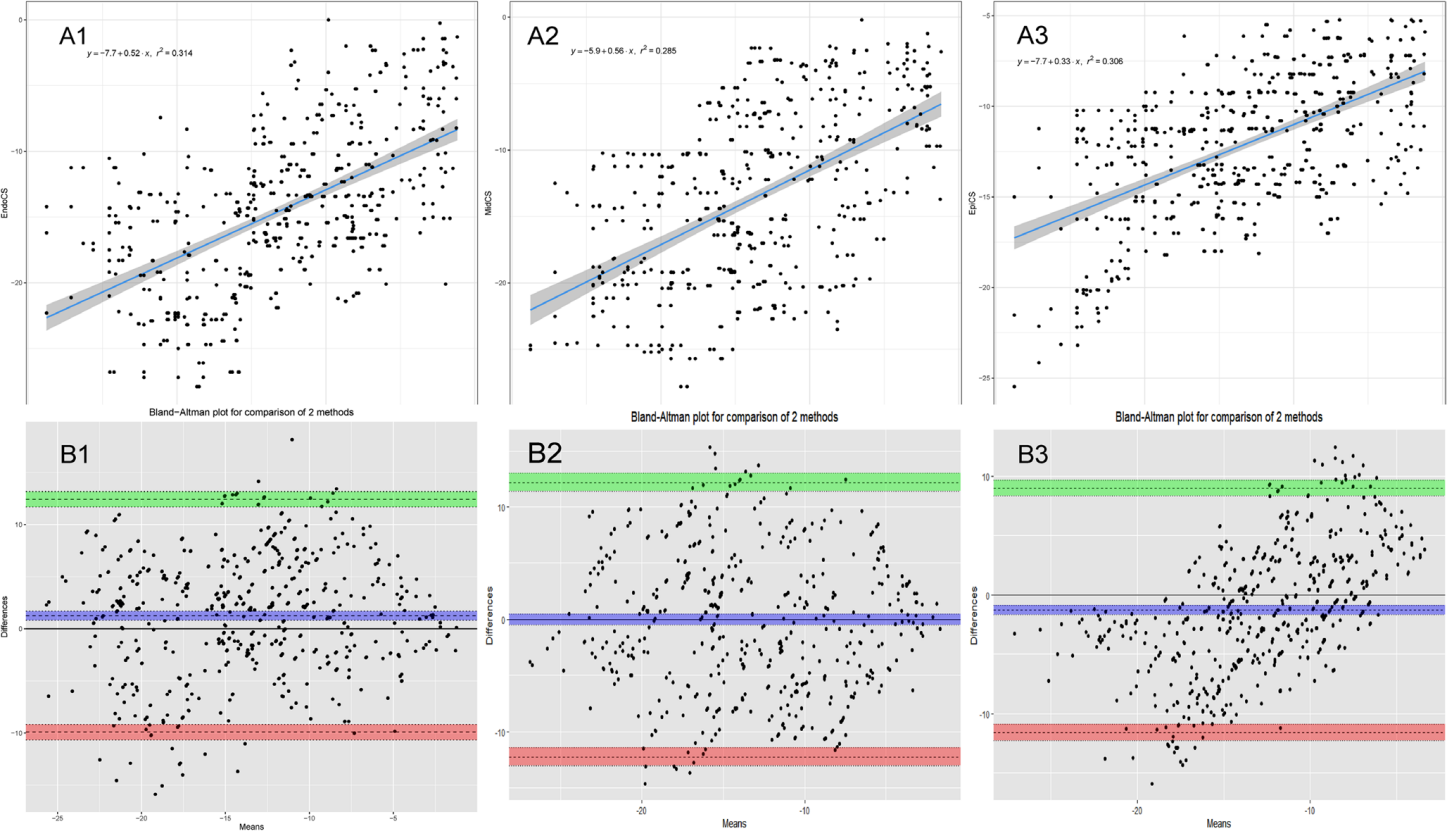
Inter-modality agreement and correlation between CMR-FT and LS-STE derived strain parameters for CS. **Notes**: **A1**: Correlation FT-CS vs. EndoCS; **B1**: Bland Altman: FT-CS vs. EndoCS; **A2**: Correlation FT-CS vs. MidCS; **B2**: Bland Altman: FT-CS vs. MidCS; **A3**: Correlation FT-CS vs. EpiCS; **B3**: Bland Altman: FT-CS vs. EpiCS

**Fig. 5:**
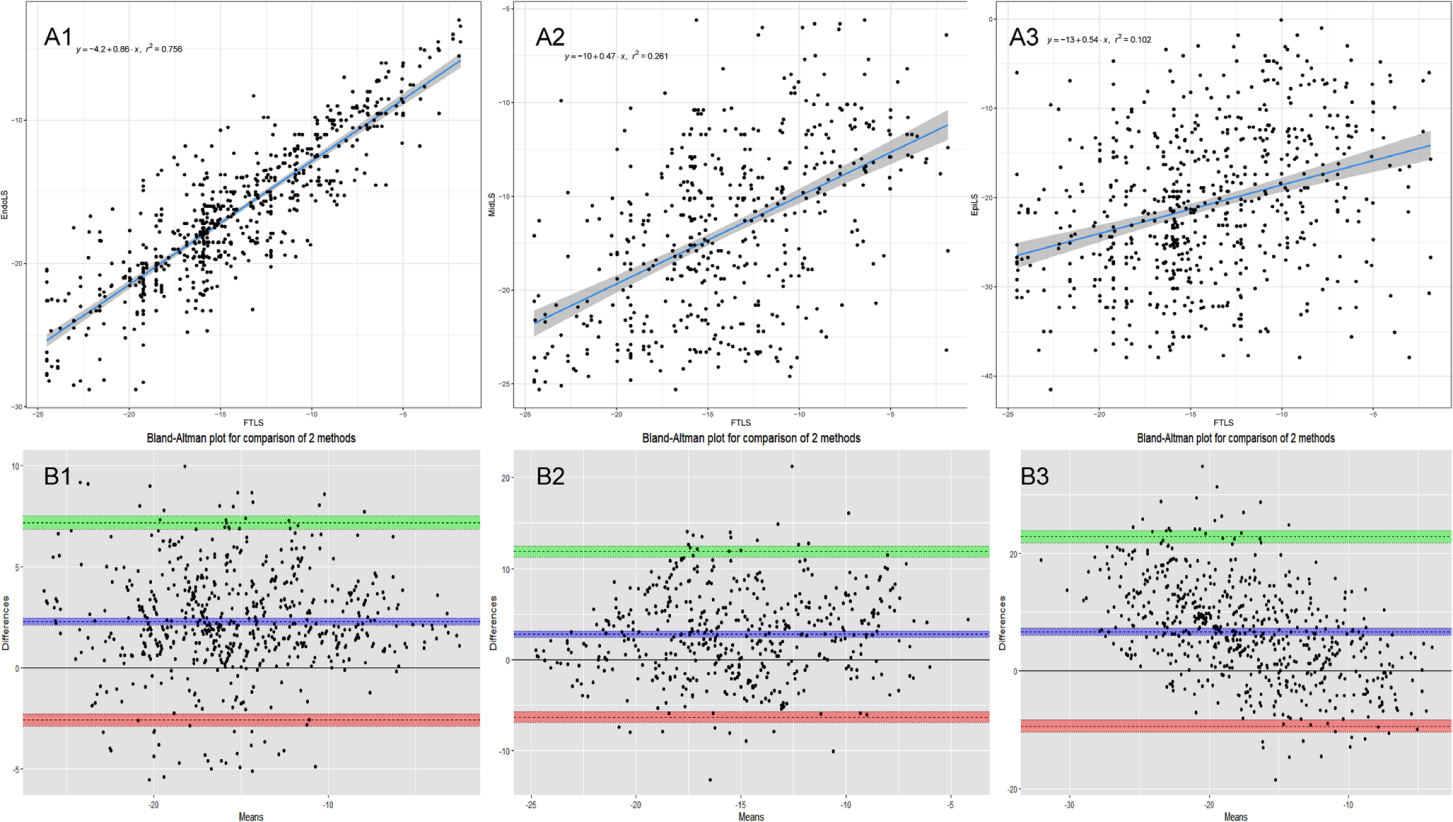
Inter-modality agreement and correlation between CMR-FT and LS-STE derived strain parameters for LS. **Notes**: **A1**: Correlation FT-LS vs. EndoLS; **B1**: Bland Altman: FT-LS vs. EndoLS; **A2**: Correlation FT-LS vs. MidLS; **B2**: Bland Altman: FT-LS vs. MidLS; **A3**: Correlation FT-LS vs. EpiLS; **B3**: Bland Altman: FT-LS vs. EpiLS

The Pearson’s correlation, intraclass correlation, and Bland-Altman analyses results between CMR-FT and LS-STE are shown in Table 2. The liner correlation and Bland-Altman analyses plots between CMR-FT and LS-STE are presented in Figs. 3, 4 and 5.

**Table 2:** Pearson’s correlation and Bland-Altman analysis between CMR-FT and LS-STE

Strain	r	ICC	P-value	Bias	LOA
FTRS vs. RS	0.33	0.44	< 0.0001	-3.26	± 21.02
FTCS vs. EndoCS	0.56	0.71	< 0.0001	1.26	± 11.16
FTCS vs. MidCS	0.53	0.70	< 0.0001	-0.02	± 12.21
FTCS vs. EpiCS	0.55	0.64	< 0.0001	-1.30	± 10.27
FTLS vs. EndoLS	0.87	0.87	< 0.0001	2.28	± 4.48
FTLS vs. MidLS	0.51	0.56	< 0.0001	2.78	± 9.10
FTLS vs. EpiLS	0.32	0.064	< 0.0001	6.68	± 16.13

**Notes**: r, Pearson’s correlation co-efficient; ICC, intraclass correlation coefficient; Bias, mean of differences between CMR-FT and STE measurements; LOA, limits of agreement, 1.96 × standard deviation of the differences between the two measurements

The corresponding values using LS-STE and FTLS were 0.87 for EndoLS vs. FTLS, 0.51 for MidLS vs. FTLS, and 0.32 for EpiLS vs. FTLS comparisons. Compared to FTLS, EndoLS for the CMVO was significantly higher than any other parameters. Agreement between the two techniques was good in a comparison of FTLS vs. EndoLS (bias = 2.28). The reliability was good between FTLS and EndoLS (ICC = 0.87), and moderate between FTLS and MidLS (ICC = 0.56).

The corresponding values for comparisons of FT-CS vs. EndoCS, FT-CS vs. MidCS, FT-CS vs. EpiCS were 0.56, 0.53, 0.55 respectively. The agreement between FT-CS and other STE-CS parameters, such as EndoCS, MidCS, and EpiCS was good (bias = 1.26, -0.02, and − 1.3, respectively). The reliability was moderate for all the three parameters (ICC = 0.71, 0.70, and 0.64, repectively).

The agreement and reliability for FTRS vs. RS was poor due to the lower correlation for RS.

### Repeatability

The results for inter- and intra- observer variability are shown in Table 3.

**Table 3:** Inter- and intra- observer variability between LS-STE and CMR-FT

			LS-STE							CMR-FT		
			RS	EndoCS	MidCS	EpiCS	EndoLS	MidLS	EpiLS	FT-RS	FT-CS	FT-LS
ICC	Inter Intra		0.97 0.97	0.92 0.99	0.97 0.98	0.97 0.99	0.93 0.98	0.93 0.98	0.93 0.97	0.98 0.92	0.99 0.99	0.96 0.99
Bias	Inter Intra		0.22 -0.03	-0.01 -0.04	0.12 -0.04	0.02 0.07	0.04 0.04	0.03 0.01	-0.01 0.14	-0.27 -0.69	0.21 0.13	0.12 -0.01
LOA	Inter Intra		± 4.35 ± 4.08	± 4.35 ± 1.78	± 3.23 ± 1.78	± 2.00 ± 0.98	± 3.40 ± 2.32	± 3.15 ± 1.85	± 5.69 ± 4.27	± 4.35 ± 7.44	± 1.65 ± 1.93	± 2.96 ± 1.70

**Abbreviations**: ICC, intraclass correlation coefficient; Bias, mean of differences between CMR-FT and STE measurements; LOA, limits of agreement; Inter, Inter- observer variability; Intra, Intra- observer variability

## Discussion

The present study’s key conclusion is that FTLS and EndoLS exhibit strong inter-modality agreement in CMVO assessment as measured using Pearson’s correlation coefficient and Bland-Altman analysis. FTLS showed better dependability compared to EndoLS. The agreement and reliability were fair in CS, while RS was inadequate for evaluating CMVO.

### Importance of CMVO evaluation

CMVO is linked with a poor prognosis, and varying degrees of CMVO occur in almost all AMI patients receiving PPCI. About 50% of patients experience spontaneous improvement over time [18].

Infarct size, along with ventricular function, clinical events, and cardiovascular mortality, is a key factor in determining a patient’s prognosis. Reversible edema, capillary damage with intramyocardial bleeding, microembolization, and several other symptoms are all present in coronary microcirculation [19]. Coronary microembolization is the AMI patient group’s most likely symptom [20]. However, during PPCI, it is unclear whether protection equipment is necessary during PPCI. In patients with acute myocardial infarction, the use of a mesh-covered embolic protection stent was superior to a standard stent [21]. Above all, numerous particle debris and a number of soluble chemicals are produced during the rupture of atherosclerotic plaque, contributing to poor microvascular perfusion. They all induced CMVO in AMI patients regardless of whether they underwent PPCI. How to measure and protect from the CMVO is an urgent problem. Heusch G et al. found that ischemic preconditioning has been used in patients with pre-infarction angina. However, it can effectively reduce the infarct size and CMVO [22]. Marcos-Garcés V et al. demonstrated that time to reperfusion, GRACE risk score, LV ejection fractions (EF) and CMVO were the novel variables for simple and straightforward major adverse cardiovascular events risk assessment (MACE) after STEMI [23].

The integrity of microcirculation structure and function after myocardial infarction is the basis of myocardial survival. The more abundant the microcirculation, the better the blood supply to the myocardium and the stronger the systolic and diastolic functional reserve. Therefore, we deduced that the strain technique has some diagnostic value in indicating the occurrence of the CMVO in the whole or segmental myocardium. Some studies based on foreign [24, 25] and Chinese [26] populations have also shown that strain analysis has good diagnostic accuracy in distinguishing non-MVO infarcts from MVO infarcts. Our team have already concluded that layer-specific analysis of 2-dimensional speckle tracking echocardiography (LS-2D-STE) can provide additional and reliable diagnostic tools to identify MVO in STEMI patients after reperfusion [27].

Due to their complicated development and high cost, invasive procedures cannot be routinely employed as the gold standard for evaluating CMVO. Safe and precise CMR-FT and LS-STE imaging methods allow for the identification of many CMVO characteristics. In CMVO, STE-derived values provide helpful diagnostic assistance. Additionally, they are significant predictors of functional prognosis in individuals with STEMI and are connected to the presence of CMVO on CMR images. To learn more about the diagnosis, prognosis, and risk stratification of STEMI with CMVO, STE analysis must be taken into account during the ultrasound examination due to its simplicity, speed of execution, low cost, and bedside feasibility [28].

### Bland-altman analysis of CMR-FT and LS-STE

Imaging can be easily performed within a routine examination for both LS-STE and CMR-FT. Hence, no extra CMR sequences are required and data analyses can be conveniently conducted using clinically approved software. Routine echocardiography is an economical and easy method to effectively assessCMVO. LS-STE can be more accurate than STE without the extra cost.

According to research by Pankaj Garg et al., CMR-FT is highly correlated with the occurrence of CMVO in STEMI patients. Compared to other CMR metrics, baseline global longitudinal peak systolic strain (GLS) showed a greater correlation with unfavorable LV remodeling [15]. It can reveal important prognostic information after myocardial infarction [29]. When compared to echocardiography, CMR-FT has already been demonstrated to provide more precise measurements of cardiac deformation without the use of a contrast agent. Circumferential peak systolic strain (CS), according to research by Torben Lange et al., is a valuable metric for describing how the distant myocardium responds [30]. According to the previous studies, CMR-FT is always used to evaluate the myocardial function, while strain values indirectly represent the compensatory capacity of the myocardial infarction. CMR-FT is also used to assess the CMVO to allow further risk stratification in patients with STEMI [10].

Good correlation was noted between CMR-FT LS and EndoLS for myocardial strain in the present study. Other LS layers showed moderate and even poor correlation. Consistent with previous studies, LS strain can be an independent predictor for MACE [31]. However, the correlation should be higher in the present study, and the result was not the same. Sun et al. showed that EndoLS was more noticeable than changes in LS strain [32]. First, the subendocardial myocardium had the most extensive damage of the three layers. Second, it could improve the correlation results because all of the segments were explored in previous studies. The present study, only focused on the CMVO segments without considering normal segments. Even though the time of evaluating STEMI patients was decreased, the accuracy of the results increased.

According to Mandoli GE and colleagues, LS-STE can be a novel, useful tool in the echocardiographic assessment of coronary microvascular disease [17]. Previous research has shown that LS-STE results are very good predictors of LV anomalies in individuals with ischemia illness. STE analysis is able to identify aberrant deformations in STEMI patients. Additionally, Mandoli discovered that CS might serve as a new echocardiographic measure for CMVO identification in LS-STE [17].

Good correlation between CMR-FT and STE-derived whole-layer GLS was also demonstrated by Pyrds et al. (n = 50), although with a lower agreement value than that shown in the Bland-Altman analysis results due to a significant bias [33]. CMR-FT generated an LS value that was lower than the EndoLS value. The CMR-FT also monitored the endocardial and epicardial borders, which both reflect the movement of intra-myocardial speckles [34]. This may be a significant reason for the decrease in CMR-FT values. However, the ICC value for EndoLS was significantly higher than that for EpiLS. Reindl, et al. already demonstrated that LS is a good parameter for CMVO detection in STEMI patients with STE and CMR-FT [35]. LS is an independent predictor for CMVO. They demonstrated that the LS is more sensitive to the myocardial disease. EndoLS has a potential application value for a risk stratification tool for STEMI patients with CMVO. Although CMR shows that CMVO is a clear sign of harm, it does not distinguish between myocardial ischemia-reperfusion injury and initial ischemia-related injury. In the present research, EndoLS may be crucial in identifying CMVO in STEMI patients with PPCI.

Moderate correlation was present between CMR-FT CS and multi-layers of CS. Mangion et al., found that CS was superior to other parameters in STE for CMVO detection, even superior to CMR-FT [36]. In the study of correlation between CMR-FT and multilayer strain, the CS with CMR-FT and LS-STE was able to provide important prognostic information for CMVO in STEMI patients. The outcomes were stable because of the disease’s transmural spread to the midmyocardium and epicardium. The outcome was more constant since the CS was more likely to be compromised in STEMI patients than other metrics. According to Leung, et al [37]. CS may be used as a diagnostic tool to evaluate CMVO in STEMI patients. The EF had an impact on the strain, particularly for LS. According to Stathogiannis, et al., [38] areas of scarring dramatically change CS. It is possible that strain from non-contrast CMR can resemble an LGE image with more tuning.

As the study highlight, the results demonstrated that CS and EndoLS can be incorporated to reliably discriminate between CMVO and the infarcted segments. The prognostic value of the multilayer strain was not reported.

Finally, the two methods’ limit of agreements (LOA) values were still wide, which was consistent with previous studies [15]. First of all, there were still a few patients in the studies and the sizeable LOA suggests that inter-modality measurements were not interchangeable [39]. Clinical decisions regarding the use of LS-STE and CMR-FT should be taken into account. Patients with poor echocardiographic image characteristics, such as reverberations, increased field noise, and poor imaging windows, may benefit from CMR-FT use. Superior image quality, broad fields of view, superior volumetric analysis, and improved tissue composition are its main advantages over STE. However, practically is CMR’s key disadvantage compared to echocardiography, which is an affordable and widely used diagnostic method. In addition, LS-STE is convenient and its accuracy is equal to that of CMR-FT. In the clinical work, STEMI patients can undergo LS-STE to assess the CMVO before PPCI, which can help to distinguish between initial ischemia and reperfusion injury.

### Limitations

First, the cvi42 measurements for CMR could only be made on the myocardial layers due to software limitations and image acquisition limitations. Second, only one software package was used to complete the strain study. Thus, repeatability between software vendors may be an issue that has to be addressed. Third, all of the patients who underwent CMR and echocardiography examinations were not evaluated on the same day due to clinical restrictions. This might have an impact on EpiLS’ connection. Furthermore, it is important to recognize that an invasive CFR and assessment of microcirculation was not performed in our study. It is also critical to note that AMI patients make up the majority of the study’s participants. Information about healthy volunteers from a larger population is still needed.

## Conclusions

The best inter-modality agreement was observed between CMR-FT and LS-STE for the measurement of CMR-FT LS and endocardial LS for all of the parameters. Bland-Altman analysis showed good reliability for EndoLS and moderate for EndoCS, MidCS, EpiCS, and MidLS.

CMVO evaluation in STEMI patients revealed that CMR-FT is a viable technique. EndoLS demonstrated good reliability for CMR-FT during CMVO evaluation using LS-STE. Three parameters in LS-STE CS were moderate compared to those in FTCS. This implies that CS may have a potential value for CMVO prognosis. In the clinical work, STEMI patients can undergo LS-STE to assess the CMVO before PPCI, which can help to distinguish between initial ischemia and reperfusion injury.

### Electronic supplementary material

Below is the link to the electronic supplementary material.

Supplementary Material 1

## Data Availability

The datasets are available by contacting the corresponding author.

## Abbreviations

CFR: Coronary flow reserve
CMR: Cardiovascular magnetic resonance
CMR-FT: Cardiovascular magnetic resonance feature tracking
CMVO: Coronary microvascular obstruction
CS: Circumferential peak systolic strain
D to W: Door to wire
EndoCS: Endocardial CS
EndoLS: Endocardial LS
EpiCS: Epicardial CS
EpiLS: Epicardial LS
GCS: Peak global circumferential
GLS: Global longitudinal peak systolic strain
ICC: Intraclass correlation coefficient
LAD: Left anterior descending
LCX: Left circumflex branch
LGE: Late gadolinium enhancement
LOA: Limits of agreements
LS: Longitudinal peak systolic strain
LS-STE: Layer-specific speckle tissue echocardiography
LV: Left ventricular
LVEF: Left ventricular ejection fraction
MidCS: Midcardial CS
MidLS: Midcardial LS
MACE: Major adverse cardiovascular events risk assessment
PCI: Percutaneous coronary intervention
PET: Positron emission tomography
PPCI: Primary percutaneous coronary intervention
RCA: Right coronary artery
ROC: Receiver-operator characteristics
RS: Radial peak systolic strain
STE: Speckle tissue echocardiography
STEMI: ST elevation myocardial infarction
