# Supplementary material for: Direct comparison of coronary microvascular obstruction evaluation using CMR feature tracking and layer-specific speckle tracking echocardiography in STEMI patients
Source: Int J Cardiovasc Imaging. 2023 Nov 13;40(2):237–47. doi: 10.1007/s10554-023-02998-5 (PMC10884157; doi:10.1007/s10554-023-02998-5)
Supplement: Supplementary file 1 — Supplementary Material 1 [file 10554_2023_2998_MOESM1_ESM.docx]

Supplementary Materials

**Figure 1**

LS-STE was performed after PPCI in a 56-year-old man with STEMI. Image analysis of longitudinal strain obtained based on the specific stratified ultrasound speckle tracking technique in a STEMI patient. The myocardial wall was divided into endocardial layer (A), mid-wall layer (B) and epicardial layer (C).


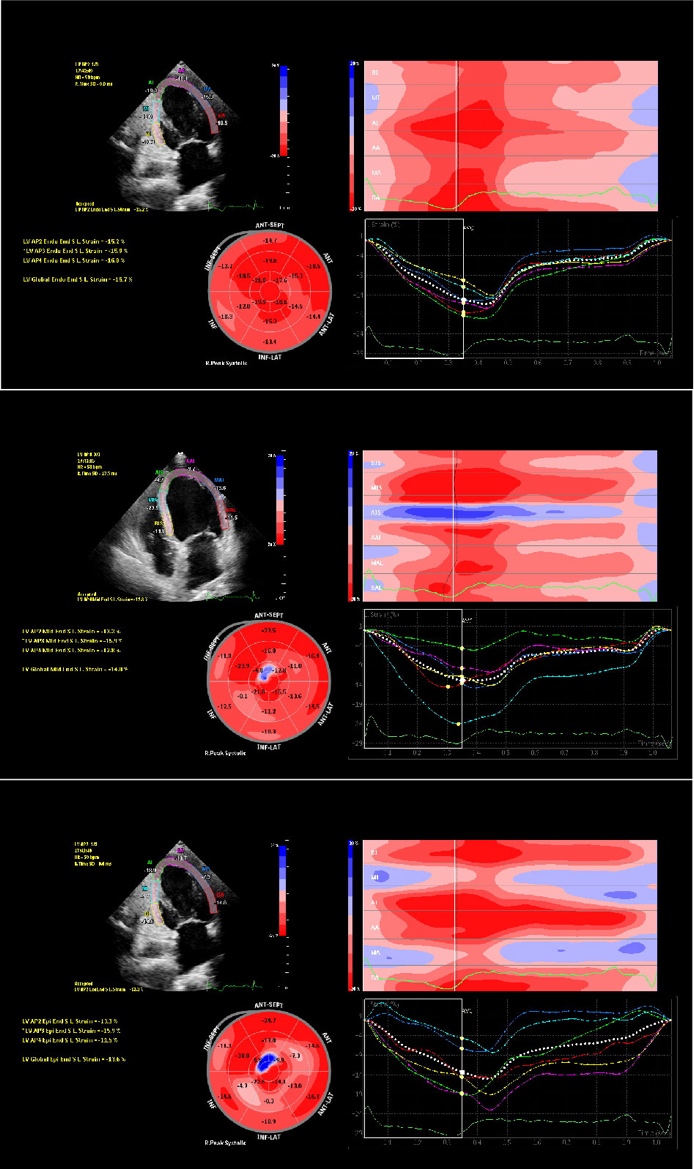


B

C

A

**Figure 2**

CMR-FT was performed after PPCI in a 56-year-old man with STEMI. In the top row are delayed enhanced postprocessing images: from left to right are apical segment, papillary muscle segment, and basal segment. The middle row shows the peak systolic strain time curves after magnetic resonance post-processing. From left to right, the curves are: circumferential strain time curve of the peak systolic period, longitudinal strain time curve of the peak systolic period, and radial strain time curve of the peak systolic period. In the bottom row are 16-segment bull's-eye views of the left ventricle obtained based on magnetic resonance feature tracking techniques, from left to right: circumferential strain, longitudinal strain, and radial strain.


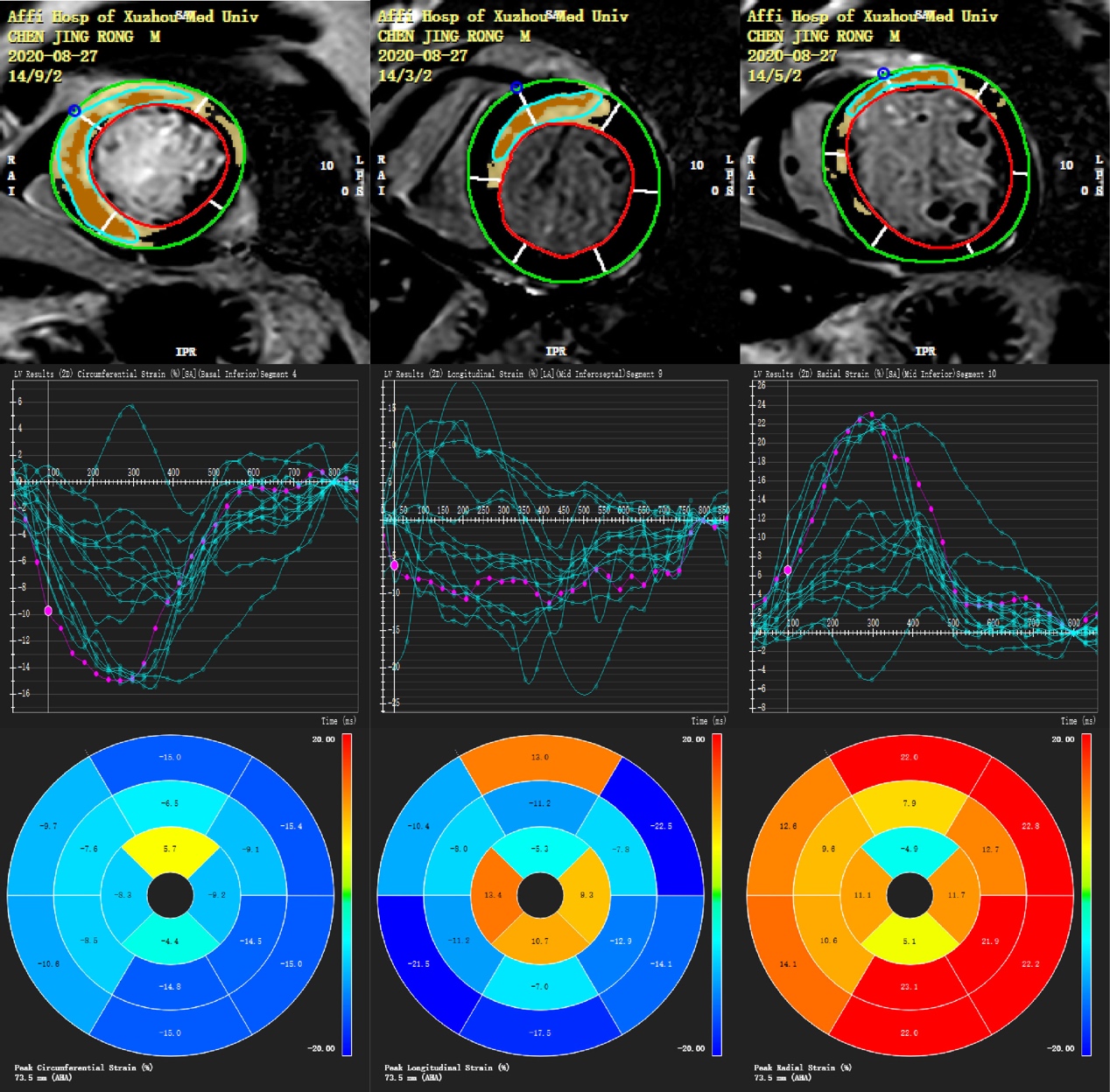


B

C
